# The influence of achievement motivation on the educational practice ability of pre-service teachers: the multiple mediating role of professional identity and learning engagement

**DOI:** 10.3389/fpsyg.2024.1463557

**Published:** 2024-09-20

**Authors:** Yanhong Peng, Cai Zhang

**Affiliations:** ^1^School of Education, Huainan Normal University, Huainan, China; ^2^Collaborative Innovation Center of Assessment for Basic Education Quality, Beijing Normal University, Beijing, China

**Keywords:** pre-service teachers, educational practice ability, achievement motivation, professional identity, learning engagement

## Abstract

**Objective:**

According to Bronfenbrenner’s bioecological model of human development, focus on exploring the mechanism of person characteristics (achievement motivation) in their own development (improvement of educational practice ability).

**Method:**

A survey was conducted on 1,225 pre-service teachers in Anhui Province, China, using the Achievement Motivation Scale, Professional Identity Scale, Learning Engagement Scale, and Educational Practice Ability Scale.

**Results:**

(1) Achievement motivation can significantly and positively predict pre-service teachers’ educational practice ability; (2) Achievement motivation can indirectly affect pre-service teachers’ educational practice ability through the mediating effects of professional identity and learning engagement; (3) Professional identity and learning engagement play a chain mediated role in the impact of achievement motivation on pre-service teachers’ educational practice ability.

## 1 Introduction

Teachers are one of the most influential and powerful forces in promoting educational equity, accessing educational opportunities, and improving educational quality, and are key to global sustainable development. Countries around the world attach great importance to improving the quality of teachers, and China regards it as the most important foundation work for building an education powerhouse strategy. Pre-service teachers are the reserve force of the teaching community, and their ability cultivation is the foundation and guarantee of teacher quality. Educational practice ability is the core manifestation of pre-service teachers’ professional competence and the key to their future competence in teaching. Exploring effective paths for cultivating and enhancing the educational practice ability of pre-service teachers is an important topic in the research field. Through literature review, it was found that existing research mainly focuses on analyzing environmental factors such as professional training objectives, training models (Fitriati et al., 2024), curriculum design (Zhang, 2020), teacher teaching philosophy and methods, attitudes and behaviors (Castaño et al., 2015), and teaching practical knowledge (Tian and Huo, 2015). Only a very small number of scholars have explored from aspects such as a reflective mindset (Goldman and Grimbeek, 2015), subjective consciousness (Bi and Qi, 2022) and personality (Biermann et al., 2015) of pre-service teachers.

Educational practice ability is the sum of psychological characteristics necessary for pre-service teachers to engage in teacher education work in the future, and is an organic unity of multiple abilities (Zhang et al., 2013). According to the bioecological model of human development by Bronfenbrenner (2001), person characteristics play an important role in their own development, which is achieved through a mechanism called the proximal processes (Bronfenbrenner and Morris, 2006). The proximal processes are a persistent interaction between the person (characteristics) and their immediate environment, and is considered to be the main driving force of individual development, influenced by both person characteristics and the environment. Bronfenbrenner described three types of person characteristics. Among them, the characteristic of force [or ‘disposition’; Bronfenbrenner (1995) is considered the most likely to influence a person’s developmental outcomes (Rosa and Tudge, 2013)]. Achievement motivation is a learned and relatively stable personality trait that serves as an internal drive to pursue and achieve goals (Wu et al., 2017). Its strength can cause individuals to have different emotional responses when facing different work or learning tasks, which can have different effects on an individual’s professional values (Yu and Huang, 2023), learning engagement (Froiland and Worrell, 2016; Cheng et al., 2022), work responsibility (Zhong et al., 2024), and the potential development of individual abilities (Ye and Kunt, 1992). Therefore, it can be seen that professional identity and learning engagement can be viewed as a sustained interaction between the person and the environment, i.e., the proximal process. Therefore, this study aims to explore the specific mechanisms by which achievement motivation (person characteristics) influences the proximal processes (professional identity and learning engagement) and promotes the improvement of pre-service teachers’ educational practice ability, providing empirical evidence for exploring effective ways to improve the educational practice ability of Chinese pre-service teachers.

### 1.1 The relationship between achievement motivation and educational practice ability

Achievement motivation is a personality psychological tendency derived from the need for achievement, which is an internal driving force that motivates individuals to be willing to engage in work they consider important or valuable, and strive for success (Feng et al., 2000). The Atkinson achievement motivation theory suggests that an individual’s achievement motivation consists of two parts: the motivation to pursue success and the motivation to avoid failure. People with high motivation to pursue success aspire to success, have confidence in doing things, are not afraid of difficulties, are brave in taking risks, dare to challenge, and are willing to unleash their abilities; People with high motivation to avoid failure tend to avoid possible failures or uncertainties, worry about failure, be easily uneasy, worry and fear (Ye and Kunt, 1992; Yang, 2013) and do not value to unleash their abilities. Therefore, individuals with stronger achievement motivation May also have stronger abilities. Qarri (2023) found through a survey of “Prenk Jakova” music high school students in Pristina that achievement motivation has a significant impact on the music skills and abilities of music high school students. The study by Zhong et al. (2024) confirmed that achievement motivation has a significant positive predictive effect on teachers’ differential teaching ability; Li (2016) found through investigation that there is a significant correlation between achievement motivation and the professional ability of educational technology interns. Although previous studies have not specifically focused on the group of pre-service teachers, nor have they specifically focused on achievement motivation and educational practice ability, pre-service teachers belong to the group of college students and are the preparatory force for the teacher group. Educational practice ability is the core component of the professional ability of normal university students. Based on this, this study proposes the first hypothesis:

*H1*: Achievement motivation has a significant positive influence on the educational practice ability of pre-service teachers.

### 1.2 The mediating role of professional identity, learning engagement, and their combination

Professional identity refers to an individual’s perception and experience of the profession they are engaged in Wang et al. (2010). It is not only a process of continuous and dynamic interaction between individuals and their professions, but also a state (Fan, 2009). It is a comprehensive professional psychological state composed of individual professional cognition, emotions, motivation, expectations, etc., closely related to their professional motivation and achievement needs. According to the basic viewpoint of achievement motivation theory, individuals with high achievement motivation have a higher pursuit of professional goals and are more likely to develop professional identity. The study by Luo et al. (2024) indicates a positive correlation between achievement motivation and professional identity among Chinese health students. In addition, professional identity can effectively promote the professional development of individuals. An individual’s positive cognition of their profession has a significant impact on their future professional motivation and professional effectiveness, and is a necessary condition for improving their professional abilities (Heinz, 2015). Sica et al. (2022) found based on survey data of undergraduate students from universities in southern Italy that professional identity is positively correlated with their planned skills. The study by Dong et al. (2024) shows a significant positive correlation between professional identity and the professional competence of university counselors. The higher the level of professional identity of a counselor, the stronger their professional ability. Wu et al. (2020) further found through investigation that professional identity is a mediating variable between achievement motivation and the development of research ability among nursing undergraduate interns. Based on the review of the above literature, this study speculates that the relationship between achievement motivation, professional identity, and individual ability May also exist in the group of pre-service teachers. Therefore, a second hypothesis is proposed:

*H2*: Professional identity plays a mediating role between achievement motivation and educational practice ability of pre-service teachers.

Learning engagement refers to the ability of students to recognize the value and significance of learning, actively and continuously participate in learning activities, not afraid of challenges and setbacks, and accompanied by positive emotional experiences, exhibiting typical characteristics of vitality, dedication, and focus (Schaufeli et al., 2002). The achievement motivation theory holds that individuals with strong achievement motivation are very active in their work and study, good at controlling themselves, trying not to be influenced by the external environment, making full use of time, and achieving excellent work and study results. Chang et al. (2022) found through a survey of graduates from two science and technology universities in northern Taiwan that achievement motivation has a positive impact on graduate students’ active learning. A survey conducted by Liu (2021) on high school students found that achievement motivation has a significant predictive effect on learning engagement. Cheng et al. (2022) found in his research that there is a significant positive correlation between academic achievement motivation and technical learning engagement among students majoring in technical subjects in physical education departments. Numerous studies have confirmed a correlation between students’ achievement motivation and learning engagement, and there are differences between different levels of achievement motivation and students’ learning engagement. In addition, learning engagement is an important indicator of individual learning adaptation and an important predictor of individual academic achievement, which has a significant impact on individual future educational opportunities, professional development, and other aspects (Jia et al., 2020; Kuncel et al., 2004). Avsec and Jagiello-Kowalczyk (2018) found that active learning is an important predictor of pre-service teacher innovation ability. Zhang’s (2017) research found that learning engagement has a significant positive influence on the professional development ability of college students. Based on this, this study speculates that achievement motivation May affect pre-service teachers’ professional abilities through learning engagement, and proposes a third hypothesis:

*H3*: Learning engagement plays a mediating role between achievement motivation and educational practice ability of pre-service teachers.

Professional identity refers to an individual’s subjective perception and attitude toward their profession (Yan, 2010). Attitude plays an important role in stimulating and guiding individual psychology and behavior, therefore, professional identity is closely related to individual behavior and has a profound impact on what individuals learn and do (Bullough and Gitlin, 2001). Individuals with high professional identity often have higher learning motivation (Zhang et al., 2016) and more self-directed learning (Xu et al., 2023). Liu et al.’s (2023) survey of Chinese engineering students showed that professional identity has a positive predictive effect on their learning engagement. Liao et al. (2024) confirmed that professional identity significantly predicts the learning engagement of vocational college students. The research by Yao and Xu (2022) and Yuan et al. (2022) found that professional identity has a significant positive impact on the learning engagement of pre-service teachers. The stronger the sense of professional identity among students, the more they engage in learning. Therefore, based on hypotheses H1, H2, and H3, this study proposes a fourth hypothesis:

*H4*: “Professional identity -learning engagement” has a chain like mediating effect between achievement motivation and educational practice ability of pre-service teachers.

**Figure 1:**
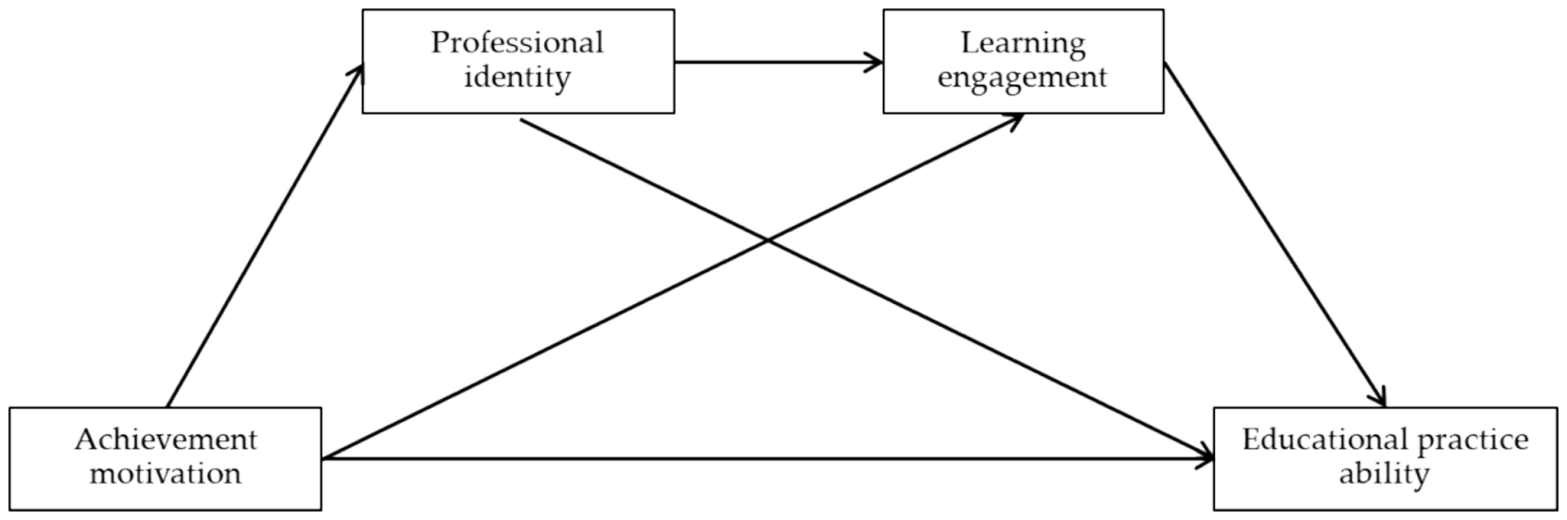
Theoretical model.

In summary, this study is based on the bioecological model of human development proposed by Bronfenbrenner, and is aimed at the pre-service teacher level in China. A model is constructed with achievement motivation as the independent variable, professional identity and learning engagement as the mediating variables, and pre-service teacher educational practice ability as the dependent variable (see Figure 1), in order to explore the impact mechanism of achievement motivation on pre-service teacher educational practice ability, and provide reference for broadening the path of improving pre-service teacher educational practice ability in China and improving the quality of pre-service teacher training.

## 2 Methods

### 2.1 Participants and procedures

This study used a convenient sampling method to select students from a normal university in Anhui Province, China. We use online questionnaires to collect data, and the data collection is conducted on an online platform.^1^ The specific data collection is implemented in two steps: one is to Targeted distribute questionnaires through the Wenjuan.com, and the other is to organize participants to answer collectively online in the school computer room. The experimenter were members of a highly trained research team. Before conducting the test, the experimenter first explain the research purpose to the subjects, emphasizing that the questionnaire is anonymous and strictly confidential, and the data obtained is only used for scientific research and not for other purposes. During the testing process, they can withdraw at any time according to their own wishes and issue an informed consent form. The subjects can resume testing after obtaining informed consent to ensure the authenticity and validity of the information filled in. Then the experimenter will explain the methods and precautions for filling out the questionnaire. Finally, read out the instructions and ask the participants to answer.

A total of 1,566 questionnaires were distributed in this study. Questionnaires with careless responses and invalid lie detection were excluded, and 1,225 valid questionnaires were retained, with an effective rate of 78.22%. Among them, 205 males (16.73%) and 1,020 females (83.27%); 424 sophomores (34.61%), 368 juniors (30.04%), and 433 seniors (35.35%); 674 students in humanities (55.02%) and 551 students in science (44.98%); 434 people in urban areas (35.43%) and 791 people in rural areas (64.57%). The mean age of the participants was 21.00 years (SD = 0.84; min = 20, max = 22).

### 2.2 Measures

#### 2.2.1 Educational Practice Ability Scale

We use a self-designed scale of educational practice ability for pre-service teachers, which was developed based on the understanding of pre-service teacher educational practice ability by Chinese and foreign scholars, as well as the professional ability standards for pre-service teachers issued by the Chinese Ministry of Education. The scale consists of 39 questions from three dimensions: teaching ability, educational ability, and reflective research ability. Among them, teaching ability includes 19 questions that describe the teaching design ability, teaching implementation ability, and teaching research ability of pre-service teachers, such as “I am able to determine appropriate classroom teaching objectives based on curriculum standards”; The ability to educate students includes 11 questions that describe the pre-service teacher’s abilities in class guidance, subject education, and activity education, such as “I have mastered the methods of providing initial guidance for student development”; The reflective research ability includes 9 questions that describe the educational and teaching research ability and reflective ability of pre-service teachers, such as: “I am able to write educational and teaching research papers or reports.” Using a five-point scoring system ranging from “1- very inconsistent” to “5- very consistent,” the higher the score, the higher the level of educational practice ability of pre-service teachers. In this study, the three dimensions of teaching ability, educational ability, and reflective research ability in the scale, as well as the overall scale were included, Cronbach’s *α* are 0.97, 0.95, 0.94, and 0.98 respectively, indicating a good fit in the construct validity index (*χ^2^/df* = 4.06; NFI = 0.94; CFI = 0.95; TLI = 0.95; SRMR = 0.01; RMSEA = 0.05).

#### 2.2.2 Achievement Motivation Scale

The Achievement Motivation Scale revised by Ye and Kunt (1992) was adopted, which consists of 30 questions in two dimensions: the motivation to pursue success and the motivation to avoid failure. Among them, the motivation for pursuing success includes 15 questions that measure the motivation related to achieving success, involving positive evaluations of the situation and expectations of the outcome, such as “I enjoy novel and difficult tasks, even willing to take risks.” The motivation for avoiding failure includes 15 questions that measure the motivation related to preventing failure, involving negative evaluations of the situation and expectations of the outcome, such as “I hate working in situations where it is completely uncertain whether failure will occur.” Using a four-point scoring system from “1- completely disagree” to “4- completely agree,” Achievement motivation score = Pursuit of success score – Avoidance of failure score. The higher the score, the stronger the achievement motivation; conversely, the weaker the achievement motivation. In this study, the two dimensions of the motivation to pursue success and the motivation to avoid failure in the scale, as well as the overall scale were included, Cronbach’s *α* are 0.86, 0.89, and 0.88, respectively.

#### 2.2.3 Professional Identity Scale

The Professional Identity Scale of pre-service teachers developed by Wang et al. (2010) was used, which was developed based on the understanding of professional identity by Chinese and foreign scholars, which not only draws on international classic structures, but also considers pre-service teachers and local characteristics in China. The scale consists of 12 questions from four dimensions: professional intention and expectation, professional volition, professional value, and professional efficacy. Among them, professional intention and expectation include three questions that describe pre-service teachers’ expectations and willingness to prepare for the teaching profession, such as “I have a willingness to communicate with frontline teachers”; Professional volition includes three questions, describing the persistence of pursuing a teaching profession in the future before graduation, such as “If there is an opportunity to choose another profession after graduation, I would still choose to be a teacher”; The professional value includes three questions, describing the value judgments of pre-service teachers toward the teaching profession and their own pre-service teacher identity, such as “I believe that teachers have a high social status”; Professional efficacy includes three questions that describe the self-efficacy of pre-service teachers in the teaching profession, such as “I believe I can become an excellent teacher.” Using a five-point scoring system from “1- completely disagree” to “5- completely agree,” the higher the score, the higher the level of professional identity of pre-service teachers. In this study, the four dimensions of Professional intention and expectation, Professional volition, Professional value, and Professional efficacy in the scale, as well as the overall scale were included, Cronbach’s *α* are 0.85, 0.70, 0.77, 0.83, and 0.87, respectively.

#### 2.2.4 Learning Engagement Scale

The Learning Engagement Scale was developed by Schaufeli et al. (2002) and revised by Fang et al. (2008), which consists of 17 questions in three dimensions: vitality, focus, and dedication. Among them, vitality includes 6 questions that describe an individual’s sustained investment of energy, time, and effort in learning tasks, such as “When I wake up in the morning, I am filled with the power of learning”; Focus includes 6 items that describe an individual’s wholehearted dedication to learning, such as “When learning, I reached a state of selflessness”; Dedication includes 5 questions that describe an individual’s belief in the significance of learning and their willingness to accept challenges in learning, such as “I think learning is valuable and meaningful.”

Using a seven-point scoring system from “1- never” to “7- always,” the higher the score, the higher the level of learning engagement. In this study, the three dimensions of vitality, focus, and dedication in the scale, as well as the overall scale were included, Cronbach’s *α* are 0.89, 0.87, 0.83, and 0.95, respectively.

### 2.3 Data analysis

This study used SPSS 26.0 to conduct descriptive statistics and correlation analysis on the data; Using Amos 26.0 to verify the validity indicators of the self-designed “Pre-service teachers Educational Practice Ability Scale”; Perform mediation analysis using the SPSS macro program PROCESS 4.1 developed by Hayes.

## 3 Results

### 3.1 Common method bias test

This study used Harman’s single factor test to test for possible common method biases. Exploratory factor analysis using SPSS 26.0 was conducted on all questions integrated into various scales, and it was found that 13 factor eigenvalues were all greater than 1. The first factor explained a variance of 29.71%, which is below the basic standard of 40%. This indicates that the common method bias of the data in this study is not significant.

### 3.2 Descriptive statistics and correlation analysis of variables

A correlation analysis was conducted between achievement motivation, professional identity, learning engagement, and the educational practice ability of pre-service teachers. The results showed that there was a significant positive correlation between achievement motivation, professional identity, learning engagement, and educational practice ability of pre-service teachers. Please refer to Table 1 for details.

**Table 1:** Description statistics and correlation relationships of each variable.

	*M*	SD	1	2	3	4
1. Achievement motivation	−0.029	0.441	1			
2. Professional identity	3.851	0.492	0.231^**^	1		
3. Learning engagement	5.434	0.754	0.474^**^	0.431^**^	1	
4. Educational practice ability	3.618	0.524	0.264^**^	0.452^**^	0.499^**^	1

^*^p < 0.05, ^**^p < 0.01.

### 3.3 The mediating role of professional identity and learning engagement

This study used the SPSS macro program PROCESS 4.1 developed by Hayes to examine the mediating effects of professional identity and learning engagement on the relationship between achievement motivation and educational practice ability of pre-service teachers, under control of grade, gender, and place of origin. Using the deviation corrected percentile Bootstrap test, with a sampling frequency of 5,000, calculate the 95% confidence interval. The results indicate (as shown in Table 2) that achievement motivation has a significant positive predictive effect on the educational practice ability of pre-service teachers (*β* = 0.267, *p* < 0.001) indicates significant overall utility. After incorporating the mediating variables of professional identity and learning engagement, the direct effect of achievement motivation on the educational practice ability of pre-service teachers is not significant (*β* = 0.029, *p* > 0.05), achievement motivation significantly positively predicts professional identity (*β* = 0.233, *p* < 0.001) and learning engagement (*β* = 0.396, *p* < 0.001), professional identity can significantly positively predict learning engagement (*β* = 0.340, *p* < 0.001) and the education practical ability of pre-service teachers (*β* = 0.279, *p* < 0.001), learning engagement significantly positively predicts the educational practice ability of pre-service teachers (*β* = 0.365, *p* < 0.001). This indicates that professional identity and learning engagement play a fully mediating role between achievement motivation and pre-service teacher educational practice ability, and a chain mediation between professional identity and learning engagement is established.

**Table 2:** Chain mediation model analysis of professional identity and learning engagement between achievement motivation and educational practice ability of pre-service teachers.

Regression equation		Overall fit index			Regression coefficient significance	
Result variables	Predictive variables	*R*	*R^2^*	*F*	*β*	*t*
Professional identity	Achievement motivation	0.243	0.059	19.137^***^	0.233	8.382^***^
Learning engagement	Achievement motivation	0.579	0.335	122.867^***^	0.396	16.466^***^
	Professional identity				0.340	14.111^***^
Educational practice ability	Achievement motivation	0.584	0.341	105.019^***^	0.029	1.091
	Professional identity				0.279	10.782^***^
	Learning engagement				0.365	12.808^***^

^*^p < 0.05, ^**^p < 0.01, ^***^p < 0.001.

**Figure 2:**
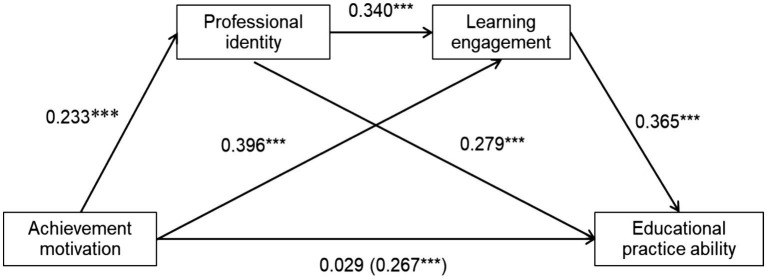
Chain mediated effect of professional identity and learning engagement on achievement motivation and educational practice ability of pre-service teachers. ^*^p < 0.05, ^**^p < 0.01, ^***^p < 0.001.

The results of the mediation effect test (as shown in Figure 2 and Table 3) indicate that the total mediation effect value of professional identity and learning engagement is 0.238, accounting for 89.139% of the total mediation model effect. The Bootstrap 95% confidence interval does not include 0, indicating that the mediation effect is significant.

**Table 3:** Bootstrap analysis of the mediating effect of the chain mediation model.

Path	Effect value	Effect proportion %	Boot CI 95%	
			Lower limit	Upper limit
Achievement motivation → educational practice ability (total effect)	0.267		0.254	0.381
Achievement motivation → educational practice ability (direct effects)	0.029	10.861	−0.027	0.096
Achievement motivation → educational practice ability (total indirect effects)	0.238	89.139	0.201	0.276
Ind1 achievement motivation → professional identity → educational practice ability	0.065	24.345	0.044	0.088
Ind2 achievement motivation → learning engagement → educational practice ability	0.144	53.933	0.117	0.174
Ind3 achievement motivation → professional identity → learning engagement → educational practice ability	0.029	10.861	0.020	0.039

The mediating effect of professional identity and learning engagement is specifically composed of the indirect effects generated by the following three paths: Ind1: the indirect effect generated by the path of achievement motivation → professional identity → educational practice ability, with an effect value of 0.065, accounting for 24.345% of the total effect of the mediating model; Ind2: the indirect effect generated by the path of achievement motivation → learning engagement → educational practice ability, with an effect value of 0.144, accounting for 53.933% of the total effect of the mediation model; Ind3: the indirect effect generated by the path of achievement motivation → professional identity → learning engagement → educational practice ability, with an effect value of 0.029, accounting for 10.861% of the total effect of the mediation model. And the Bootstrap 95% confidence intervals for all three paths do not include 0, indicating that the mediating effects of all three paths are significant.

## 4 Discussion

### 4.1 The influence of achievement motivation on educational practice ability

Research has found that achievement motivation has a significant positive predictive effect on the educational practice ability of pre-service teachers. Research hypothesis 1 has been validated and is generally consistent with the research findings of Qarri (2023) and Li (2016). The educational practice ability of pre-service teachers is related to their achievement motivation. The stronger the achievement motivation, the higher the level of educational practice ability of pre-service teachers. Mcclelland’s (1975) theory of social achievement motivation suggests that individuals with stronger needs for achievement motivation tend to perform more positively in their actions. Achievement motivation is the internal force that drives individuals to tirelessly strive to maximize their self-worth (Mcclelland, 1975). Pre-service teachers will encounter many learning tasks with different levels of difficulty during their growth process. Pre-service teachers with strong achievement motivation are more eager to achieve success and tend to accept more challenging tasks. They view learning challenges as opportunities for self-worth realization and actively respond to them. The likelihood of completing learning tasks with high quality will increase. In the process of completing tasks, the knowledge learned will be applied in practice, skills will be fully honed, and their abilities will also be significantly improved (Feng, 2013).

### 4.2 The independent mediating role of professional identity

This study found that professional identity plays a mediating role between achievement motivation and pre-service teacher educational practice ability. Hypothesis 2 of the study was validated, which is basically consistent with the research results of Wu et al. (2020). Professional identity not only plays a mediating role in the influence of achievement motivation on the research ability of medical students, but also serves as an important mediating variable between achievement motivation and the educational practice ability of pre-service teacher in educational research. Specifically, achievement motivation cannot only directly affect the educational practice ability of pre-service teachers, but also indirectly influence their educational practice ability through professional identity. Achievement motivation is a social motivation, which is an internal driving force for individuals to strive for excellence in order to achieve higher goals (Yuan, 2020). Individuals with high achievement motivation have a clearer understanding of their own goals (Wang et al., 2022) and a more mature understanding of their professions (Wang et al., 2015). To meet social expectations, gain social recognition and approval, one can be more proactive in making their behavior more in line with social requirements (Zhang, 2005). Therefore, pre-service teachers with stronger achievement motivation are more inclined to view their teaching work as an important way of self-realization, and their goal of becoming a socially recognized excellent teacher is clearer and their desire is stronger. This desire will promote their internalization of professional identity and norms (Porteous and Machin, 2018), enhance their positive perception and experience of the teaching profession, and improve their sense of identity with the teaching profession. Pre-service teachers with a stronger sense of professional identity have a deep understanding and recognition of the teaching profession they will engage in and their current pre-service teacher identity (Izadinia, 2013; Trent, 2011; Luo et al., 2024; Zhu and Wang, 2017), the stronger the willingness to engage in the teaching profession (He et al., 2022), and the more positively one can view the teaching profession, overcome difficult learning environments, and focus on learning and improving professional skills (Abednia, 2012), better grasp the necessary knowledge and educational teaching abilities for the teaching profession, and prepare for future competent teaching work.

### 4.3 The independent mediating role of learning engagement

This study found that achievement motivation can indirectly affect pre-service teachers’ educational practice ability through learning engagement. Hypothesis 3 was validated, which is consistent with the research results of Liu (2021) and Zhang (2017).

On the one hand, achievement motivation is an important psychological driving factor in personality that affects an individual’s level of effort (Yang et al., 2016), and has a significant promoting effect on students’ learning. Individuals with high achievement motivation often have higher achievement goals and successful experiences, and become more optimistic and positive in the process of striving toward their goals (Wang et al., 2022), reducing the perception of academic fatigue (Moghadam et al., 2020), enhance academic confidence (Chang et al., 2022), so as to be more proactive and actively engaged in learning. Therefore, students with stronger achievement motivation have more learning engagement. On the other hand, learning engagement is an important manifestation of students’ initiative and level of effort in participating in learning activities (Zhu, 2022) and it is also an important influencing factor in the development of students’ abilities. The educational practice ability of pre-service teachers is a necessary professional core ability for them to be competent in teaching work in the future, and its formation and development are closely related to the learning of educational knowledge and skills. Pre-service teachers with stronger achievement motivation are more proactive in learning educational knowledge and work harder in learning and training educational skills, which can better improve their educational practice abilities. Moreover, the improvement of educational practice ability brought about by learning engagement will give pre-service teachers a sense of success, stimulate their more learning engagement, and further promote the development of their educational practice ability.

### 4.4 The chain mediating role of professional identity and learning engagement

This study also found that achievement motivation can indirectly affect the educational practice ability of pre-service teachers through the mediating chain of professional identity and learning engagement, which verifies research hypothesis 4. Previous studies have shown that achievement motivation is an important antecedent variable for professional identity. Achievement motivation can promote pre-service teachers to have a positive view of the teaching profession they will engage in, and enhance their sense of identity with the teaching profession. The social identity theory holds that when an individual adopts the membership qualifications of a certain social group to establish their social identity, they will endow themselves with characteristics that conform to the internal group. Therefore, individuals with a strong professional identity will endow themselves with the characteristics of the professional group they identify with, and have more motivation to improve their professional skills through learning and training (Dong et al., 2024). The stronger the professional identity of pre-service teachers, the stronger their learning dynamic, and thus they are more enthusiastic, focused, and hardworking in their professional knowledge learning and related professional skills training (Lin, 2019), gradually improving their professional abilities through systematic learning activities and solid skills training (Wang, 2018).

### 4.5 Implications and limitations

This study is based on Bronfenbrenner’s theory of bioecology of human development, and verifies the mechanism by which person characteristics promote their own development by influencing proximal processes. This study found that achievement motivation can directly predict pre-service teachers’ educational practice ability, but more indirectly through the mediating effect of professional identity and learning engagement. The results of this study provide a practical reference path for teacher training institutions to improve the educational practice ability of pre-service teachers. Research enlightenment: It is necessary to enhance achievement motivation and provide internal motivation for improving the educational practice ability of pre-service teachers. Strengthening professional identity and laying a solid foundation for enhancing the educational practice ability of pre-service teachers. Enhance learning engagement and inject lasting strength into enhancing the educational practice ability of pre-service teachers.

There are also some shortcomings in this study. Our research findings are based on a survey of pre-service teachers in China, and their promotion May be limited. Based on cross-sectional data, the study only provides a static snapshot of the achievement motivation, professional identity, learning engagement, and educational practice ability level of pre-service teachers, which cannot reflect the dynamic development process of their educational practice ability over time. Future research can supplement the tracking survey of pre-service teachers, examining the dynamic process of their educational practice ability over time, influenced by achievement motivation, professional identity, and learning engagement, further enriching the research on the mechanism of the influence of pre-service teacher educational practice ability.

## 5 Conclusion

The following conclusions are drawn from this study: (1) achievement motivation can significantly positively predict the educational practice ability of pre-service teachers; (2) professional identity and learning engagement play a mediating role in the influence of achievement motivation on the educational practice ability of pre-service teachers; (3) professional identity and learning engagement have a chain like mediating effect between achievement motivation and educational practice ability of pre-service teachers.

## Data Availability

The original contributions presented in the study are included in the article/supplementary material, further inquiries can be directed to the corresponding author.

## Footnotes

^1^https://www.wenjuan.com/
